# Sepsis-Associated Encephalopathy: A Mini-Review of Inflammation in the Brain and Body

**DOI:** 10.3389/fnagi.2022.912866

**Published:** 2022-05-27

**Authors:** Hiroshi Ito, Sanae Hosomi, Yoshihisa Koyama, Hisatake Matsumoto, Yukio Imamura, Hiroshi Ogura, Jun Oda

**Affiliations:** ^1^Department of Traumatology and Acute Critical Medicine, Graduate School of Medicine, Osaka University, Osaka, Japan; ^2^Department of Neuroscience and Cell Biology, Osaka University Graduate School of Medicine, Osaka, Japan; ^3^Addiction Research Unit, Osaka Psychiatric Research Center, Osaka Psychiatric Medical Center, Osaka, Japan

**Keywords:** sepsis-associated encephalopathy, hippocampus, interleukin-1β, delirium, dementia

## Abstract

Sepsis is defined as a life-threatening multi-organ dysfunction triggered by an uncontrolled host response to infectious disease. Systemic inflammation elicited by sepsis can cause acute cerebral dysfunction, characterized by delirium, coma, and cognitive dysfunction, known as septic encephalopathy. Recent evidence has reported the underlying mechanisms of sepsis. However, the reasons for the development of inflammation and degeneration in some brain regions and the persistence of neuroinflammation remain unclear. This mini-review describes the pathophysiology of region-specific inflammation after sepsis-associated encephalopathy (SAE), clinical features, and future prospects for SAE treatment. The hippocampus is highly susceptible to inflammation, and studies that perform treatments with antibodies to cytokine receptors, such as interleukin-1β, are in progress. Future development of clinically applicable therapies is expected.

## Introduction

Sepsis is defined as a life-threatening multi-organ dysfunction triggered by an uncontrolled host response to infectious disease. Systemic inflammation elicited by sepsis can cause acute cerebral dysfunction, characterized by delirium, coma, and cognitive dysfunction, known as septic encephalopathy. Septic encephalopathy develops in 53% of patients with sepsis (Sonneville et al., 2017). In addition, septic encephalopathy is associated with increased mortality and with a 2.22-fold increased risk of developing dementia in the long term (Eidelman et al., 1996; Fritze et al., 2020). Septic encephalopathy is considered a diffuse brain dysfunction resulting from a systemic inflammatory response to infection, and not a symptom of direct central nervous system infection (Iacobone et al., 2009; Gao and Hernandes, 2021; Gu et al., 2021; Manabe and Heneka, 2021). Moreover, septic encephalopathy is an extremely critical condition that affects the functional prognosis and life expectancy of septic patients, and there is a strong need to elucidate and control its mechanisms.

Regarding the underlying mechanisms of sepsis, recent evidence suggests that endothelial cell degeneration, enhanced blood-brain barrier (BBB) permeability, and tight junction protein loss promote and trigger the influx of inflammatory mediators, such as interleukin (IL)-1β and tumor necrosis factor (TNF)-α, into the brain (Gao and Hernandes, 2021; Gu et al., 2021; Manabe and Heneka, 2021). However, the reasons why some brain regions develop inflammation and degeneration and the factors that determine the persistence of neuroinflammation remain unclear.

This mini-review describes the pathophysiology of region-specific inflammation after SAE, clinical features, and therapeutic measures attenuating SAE.

## Characteristics of Septic Encephalopathy: A Region-Specific Perspective

### Clinical Features in the Acute Stage of Sepsis

Acute and reversible deterioration of mental status is often associated with sepsis, and this disorder may lead to SAE. Diffuse cerebral dysfunction due to a systemic inflammatory response to infection is considered SAE, which does not include direct central nervous system infection (Iacobone et al., 2009). SAE is associated with abnormal biomarkers, such as elevated IL-1β levels, and by neuroradiologic imaging findings, such as hippocampus atrophy on brain magnetic resonance imaging (MRI). Moreover, it is assessed by various methods to identify which of the brain areas are affected (Table 1).

**TABLE 1:** Brain dysfunction in patients with sepsis.

Model	Region	Acute or chronic phase	Characteristics	Symptoms	References
Human (sepsis)	White matter	Acute phase	MRI revealed leukoencephalopathy and ischemic stroke. There was an association between imaging findings and DIC and mortality.		Polito et al., 2013
Human (sepsis)	Cerebral cortex, hippocampus, cerebral white matter	Acute phase	MRI revealed decreased volume compared to healthy controls.		Orhun et al., 2020
Human (sepsis)	Frontal lobe, hippocampus	Chronic phase (after 3 and 12 months)	Hippocampal volume at discharge with long delirium decreased on MRI evaluation; volume of superior frontal lobe decreased after 3 months.	Decreased executive function was associated with a decrease in the superior frontal lobe volume.	Gunther et al., 2012
Human (sepsis)	White matter	Chronic phase	White matter disruption on MRI evaluation was associated with longer delirium period.	Dementia after 12 months was associated with white matter disruption.	Morandi et al., 2012
Human (sepsis)	Frontal junctional cortex, lenticular nuclei, dentate nucleus, medullary olive	Acute phase	Pathological evaluation showed ischemic damage, which was a likely sign of apoptosis of neurons.		Sharshar et al., 2003
Human (sepsis, pneumonia)	Medial temporal lobe, cortical region	Chronic phase (after 12–18 months)		Pattern and spatial recognition memory impairment. Dysfunction of parahippocampal complex.	Andonegui et al., 2018

*MRI, magnetic resonance imaging; DIC, disseminated intravascular coagulation.*

In patients with septic shock who develop acute brain dysfunction, leukoencephalopathy (confluent or diffuse white matter lesions), and ischemic stroke can be detected on magnetic resonance imaging (MRI) (Polito et al., 2013). Ischemic stroke is associated with disseminated intravascular coagulation and increased mortality (Polito et al., 2013; Iba et al., 2019). The volumes of the cerebral cortex, hippocampus, amygdala, and cerebral white matter were smaller in patients with SAE than in healthy controls (Orhun et al., 2020). Among the non-survivors of SAE, the volume of the hippocampus was smaller than that of healthy controls and SAE survivors (Orhun et al., 2020).

Tissue samples from patients with delirium showed activated microglia and astrocytes and elevated levels of IL-6 in the hippocampus. This condition is associated with delirium in older patients, suggesting a relationship between delirium and inflammatory mechanisms (Munster et al., 2011).

### Basic Studies on the Acute Phase of Sepsis

Various studies using mouse models of sepsis have been conducted on multiple brain regions (Table 2). In an *in vivo* study, microglia in the hippocampus showed morphological changes at 6 h after the systemic administration of lipopolysaccharide (LPS), which is a characteristic of activation. Staining for ionized calcium-binding adaptor molecule 1 (Iba1), a protein expressed in microglia and macrophages and a cytoskeletal component involved in migration, increased (Johansson et al., 2013). In previous works, the microglia and astrocytes were found to be activated in the cortex and hippocampus. Apoptosis of neurons in the cortex and hippocampus resulted in a decrease in the number of neurons (Lee et al., 2008; Semmler et al., 2008). In one such study, activation of microglia in the hippocampus in a mouse model of CLP was reported to be stronger than that in the cortex. This suggests that microglial activation by sepsis is different in a region-specific manner and that the hippocampus is more affected by sepsis (Moraes et al., 2015).

**TABLE 2:** Brain dysfunction in mouse and rat sepsis models.

Model	Region	Acute or chronic phase	Characteristics	Symptoms	References
Mouse (LPS model)	Hippocampus	Acute phase (after 6 h)	Microglial processes thickened and Iba1 staining increased.		Johansson et al., 2013
Mouse (LPS model)	Hippocampus	Acute phase (after 2 h)	mRNA of IL-1β increased.	Hypothermia after LPS or IL-1β administration. Locomotor activity was reduced after IL-6, IL-1β, and LPS administration.	Skelly et al., 2013
Mouse (LPS model)	Hippocampus	Acute phase (after 6 h)	mRNA of IL-1β and IL-6 increased.	IL-1 receptor antagonist treatment decreased hippocampal microglia and improved cognitive function.	Terrando et al., 2010
Mouse (LPS model)		Acute phase (3–9 h)		LPS treatment caused impaired memory retention. IL-1β antagonist treatment ameliorated cognitive dysfunction.	Skelly et al., 2019
Mouse (LPS model)	Hippocampus	Chronic phase (after 7 and 63 days)	Excitatory synapses decreased by microglia.		Manabe et al., 2021
Mouse (LPS model)	Frontal cortex, hippocampus, cerebellum	Chronic phase (after 2 months)	LPS-treated mice showed sustained microglial activity and increased mRNA for IL-1β and TNF-α compared to NOS2 KO mice.	LPS-treated mice had cognitive deficits; LPS administration to NOS2 KO mice did No change in the cognitive deficits.	Weberpals et al., 2009
Mouse (LPS model)	Hippocampus	Chronic phase (after 7 days)		BDNF inhibitor treatment improved memory impairment and LTP.	Hippensteel et al., 2019
Mouse (LPS model)	Hippocampus	Chronic phase (after 7 days)	Administration of sulindac sulfide suppressed amyloidogenesis and neuronal cell death.	Memory impairment caused by LPS. Memory impairment improved after treatment with sulindac sulfide.	Lee et al., 2008
Mouse (LPS model)	Hippocampus	Acute phase	IL-1β treatment decreased membrane potential and induced apoptosis.		Skelly et al., 2019
Mouse (CLP model)	Hippocampus	Acute phase (after a few days)	Nox2 expression was inhibited after the administration of NADPH oxidase inhibitor.	Cognitive impairment was improved 15 days after NADPH oxidase inhibitor administration.	Hernandes et al., 2014
Mouse (pneumonia model)	Frontal lobe	Acute phase (after 1 day)	Neutrophils adhered to the blood vessels in the frontal lobe.	Dementia after 14 days	Andonegui et al., 2018
Mouse (influenza infection model)	Hippocampus	Chronic phase (after 30 days)	Dendritic density decreased.	Spatial memory formation was impaired.	Hosseini et al., 2018
Mouse (IL-1R1 reporter mouse line)	Hippocampus	Acute phase (immediately after IL-1 administration)	Stimulation of hippocampal endothelial cells with IL-1 induced IL-1 from microglia.		Liu et al., 2019
Rat (LPS model)	Cortex, hippocampus	Acute phase (after 1 day)	Decreased brain glucose uptake in neocortical regions. Neuronal decrease in the cerebral cortex and hippocampus.		Semmler et al., 2008
Rat (LPS model)	Hippocampus, midbrain, cerebellum	Acute phase (after 24 h)	iNOS inhibitor treatment decreased apoptotic cells and Bcl-2 positive cells in the hippocampus.		Semmler et al., 2005
Rat (LPS model)	Hypothalamus, hippocampus	Acute phase (2 h)	IL-1 mRNA, protein expression increased.		Hosoi et al., 2000
Rat (CLP model)	Prefrontal cortex, hippocampus, striatum	Acute phase (after 24 h)	Treatment with IL-1β receptor antagonist improved BBB permeability and suppressed elevated IL-1β, TNF-α, and IL-6 levels.	IL-1β receptor antagonist treatment improved cognitive dysfunction after 10 days.	Mina et al., 2014
Rat (CLP model)	Hippocampus	Chronic phase (after 10 days)	Minocycline treatment reduced cytokine levels and BBB breakdown in the hippocampus.		Michels et al., 2015
Rat (intraperitoneal injection of E-coli)	Hippocampus	Chronic phase	Aging rats had elevated IL-1 protein levels in the hippocampus compared to young rats.		Barrientos et al., 2006

*LPS, lipopolysaccharide; Iba1, ionized calcium-binding adapter molecule 1; IL-1β, interleukin-1β; TNF, tumor necrosis factor; NOS2 KO mice, nitric oxide synthase 2 knockout mice; BDNF, brain-derived neurotrophic factor; LTP, long-term potentiation; CLP, cecal ligation and puncture; BBB, blood-brain barrier.*

In addition to these morphological changes, there were region-specific features related to cytokine expression. Under physiological conditions, brain IL-1 receptor 1 (IL-1R1) was expressed primarily in the choroid plexus and endothelial cells and rarely in astrocytes but was not expressed in the microglia.

In the microglia, where IL-1R1 expression was not originally found, IL-1ß mRNA was expressed when endothelial cells were stimulated with IL-1ß. In addition, IL-1R1 expression in the neurons was most strongly confirmed in the hippocampus, where multiple cell types expressing IL-1R1 mRNA have been identified in endothelial cells, astrocytes, ependymal cells, and choroid plexus cells (Liu et al., 2019). Peripheral administration of IL-1β and TNF-α significantly increased hippocampal IL-1β mRNA expression (Skelly et al., 2013), while a 6.5-fold increase in IL-1β and a 15-fold increase in IL-6 mRNA expression were noted in the hippocampus at 6 h after LPS administration (Terrando et al., 2010). IL-1β is secreted from microglia activated by LPS and can inhibit synaptogenesis (Moraes et al., 2015).

In another study, positron emission tomography revealed that in the LPS model mice, regional cerebral blood flow (rCBF) was reduced in the cerebral cortex and alpha activity on electroencephalography decreased. In addition, cerebral glucose uptake was maintained in the hippocampus and thalamus but decreased in all neocortical regions (Semmler et al., 2008). These findings suggested that there were differences in the regional vulnerability among brain areas.

In a report on functional impairment, locomotor activity clearly decreased at 2 h after administration of systemic IL-6, IL-1β, and LPS. The central nuclei of the amygdala are activated, and the frontal cortex is affected, which is thought to be associated with symptoms, such as increased anxiety, depression, and delirium (Skelly et al., 2013). Dysfunction of working memory is induced by LPS *via* circulating IL-1β. In addition, direct action of IL-1β on the hippocampus may result in neuronal dysfunction and promote neuronal cell death (Skelly et al., 2019). Indeed, the IL-1β level was found to be elevated on day 9 after CLP. Especially, IL-1β inhibited synaptogenesis, with excitatory synapses in the hippocampus being reduced on day 9. In behavioral experiments, the cognitive index also decreased on day 9, consistent with the symptom course (Moraes et al., 2015).

### The Clinical Features in the Chronic Stage of Sepsis

The clinical profile of septic encephalopathy has been reported not only in the acute phase but also in the chronic phase. Sepsis survivors often exhibit prolonged cognitive impairment (Fritze et al., 2020).

Behavioral tests performed on patients recovering from sepsis due to pneumonia showed specific cognitive impairments in visuospatial memory function. This pattern of impairment suggests the possibility of dysfunction in the parahippocampal complex, which is highly dependent on environmental perturbations (Andonegui et al., 2018).

There are also reports on the associations among delirium duration, imaging findings, and clinical symptoms. Longer duration of delirium was related to smaller hippocampal volumes on computed tomography (indicative of hippocampal atrophy) at discharge. In addition, volume reduction in the superior frontal lobes was observed on follow-up scan at discharge and at 3 months later. Moreover, patients with reduced superior frontal lobe volume displayed reduced executive function and visual attention at 3 months after discharge (Gunther et al., 2012). MRI examinations also showed that the duration of delirium in the intensive care unit (ICU) caused white matter disruption on CT at discharge and at 3 months later; similarly, worse cognitive scores assessed by the Repeatable Battery for the Assessment of Neuropsychological Status at 12 months was associated with white matter disruption (Morandi et al., 2012).

### Basic Studies on the Chronic Phase of Sepsis

Studies have been published on changes in neural tissue in the chronic phase using a sepsis model. In one such study, infection with influenza virus led to a reduction in dendritic density in the hippocampal subregions, both in the superior and inferior DG at 30 days after infection. With this reduction in density, activated microglia increased (DG-superior: 49.18%, DG-inferior: 55.96%) (one-way analysis of variance *F*_*DG–superior(*3_,_76)_ = 17.57, *p* < 0.0001, and *F*_*DG–inferior(*3_,_76)_ = 22.16, *p* < 0.0001), long-term potentiation (LTP) in the hippocampus decreased, and spatial memory formation was impaired. This finding indicates that inflammation caused by the influenza virus resulted in functional and structural alterations in the hippocampal network (Hosseini et al., 2018).

Subsequent studies have reported on mechanisms that result in functional and structural changes in the hippocampal network. In the LPS mouse model, no significant effects were observed in neurons and synapses at 7 and 63 days post-infection. However, in the CA3 region of the hippocampus, excitatory synapses and complement factor 3 punctuation showed a delayed, region-specific decrease at 63 days. This was associated with elevated CD11b (a marker of microglia or macrophages) levels in microglia for more than 1 week after LPS administration (*E. coli* LPS: 170.16 ± 3.99%, *E. coli* LPS vs. Veh: *p* < 0.0001), indicating that microglia were not affected until 63 days after infection by hippocampal CA 3 synapses involved in the loss of synapses (Manabe et al., 2021). Loss of synapses over a prolonged period after inflammation may have a functional impairment effect.

LPS administration to wild-type mice significantly increased the TNF-α and IL-1β mRNA levels in the cortex and hippocampus, as well as the TNF-α mRNA levels in the cerebellum (Weberpals et al., 2009). Treatment of aging mice with LPS significantly increased the hippocampal IL-1β levels compared to those of young mice, in addition to age-related effects on cognitive function (Barrientos et al., 2006).

The hippocampus is affected in a region-specific manner by blood factors, resulting in hippocampal dysfunction. In sepsis, the endothelial glycocalyx is degraded, and heparan sulfate fragments are released into the circulatory system. Heparan sulfate is sufficiently large and sulfated to bind to brain-derived neurotrophic factor (BDNF), and circulating heparan sulfate fragments enter the hippocampus. Heparan sulfate inhibits BDNF and is hypothesized to block BDNF-specific functions related to spatial memory formation. To examine these issues, Hippensteel et al. examined N- and 2-O-sulfated heparan sulfate and cognitive impairment in sepsis and found that the presence of circulating 2S-rich BDNF-avid heparan sulfate fragments at the onset of sepsis was associated with cognitive impairment by 2 weeks after ICU discharge. The entry of circulating heparan sulfate fragments into the hippocampus sequesters BDNF, impairs LTP, and causes septic cognitive impairment (Hippensteel et al., 2019).

## Discussion

### Why Is Region-Specific Inflammation Observed?

Some studies (Yokota et al., 2003; Fong et al., 2006) have reported a reduction in rCBF during acute delirium in the frontal, parietal, and occipital lobes. Reduced cerebral blood flow, if sufficiently persistent, can lead to cell death, neuronal loss, and brain atrophy (Sharshar et al., 2004; Semmler et al., 2005; Gunther et al., 2012).

In general, local inflammation is the response of tissues to infection and damage. In the acute phase, dilation of vessels helps transport cells and molecules to the site of infection or damage to elicit a protective response. However, sepsis induces systemic hypotension, resulting in reduced cerebral perfusion. In addition, in the ICU, sedation worsens the situation (Egi et al., 2021). Especially in older adults, age-related decline in cardiovascular function may impair cerebral blood flow regulation, leading to the disruption of neuronal micro-environmental homeostasis (O’Rourke and Hashimoto, 2007).

Systemic inflammation, such as sepsis, produces nitric oxide (NO), which plays a key role in endotoxin-induced vasodilation and myocardial dysfunction. Endothelial injury results in microvascular thrombosis, vasodilation, and hypotension, which lead to tissue hypoxia and multi-organ dysfunction (Reinhart et al., 2002). Similar processes may occur in the brain, and if left untreated for an extended period, they can lead to neuronal cell death and brain atrophy (Sharshar et al., 2004). There is also a significant correlation between reduced cerebral blood flow, reduced glucose metabolism in the brain, and reduced neuronal cell death and neural activity (Semmler et al., 2008). Thus, septic encephalopathy involves cell death due to reduced cerebral blood flow and inflammation. Therefore, regions susceptible to ischemia, for example, lenticular nuclei, and medullary olives, are more likely to be damaged and may have impaired region-specific functions. However, the effects of septic inflammation are more significant than those of ischemia. A postmortem study suggested that during septic shock, autonomic neurons, including the amygdala, in the brain are subject to apoptosis by NO rather than ischemia (Sharshar et al., 2003).

In various experimental sepsis models, the complexity of inflammation and apoptosis-promoting factors and increased microglial activation and oxidative stress led to hippocampal destruction (Semmler et al., 2008; Zaghloul et al., 2017; Peng et al., 2018; Fu et al., 2019). Microglial activation has also been associated with reduced hippocampal volume and cognitive dysfunction in Alzheimer’s disease and other diseases (Orhun et al., 2020). Thus, the hippocampus is susceptible to inflammation due to its function. Stimulation of the vagus nerve promotes IL-1β transcription in the hippocampus, which improves memory formation and maintenance (Hosoi et al., 2000). The hippocampus is responsible for the majority of learning and memory processes, and IL-1 receptors are most highly expressed in the hippocampus. Accordingly, it has been reported that the hippocampus is highly susceptible to the unfavorable effects of neuroinflammation (Parnet et al., 1994; Gemma et al., 2005; Mina et al., 2014).

Area-specific symptoms are also observed in the chronic phase even after the acute inflammation subsides, possibly because activated microglia persist in the chronic phase, causing brain atrophy due to neuronal degeneration (Manabe et al., 2021).

### Future Treatments

The pathophysiology of septic encephalopathy is becoming clearer, and studies have shown that knockout mice or antagonists are administered with several factors that affect the pathophysiology. In this issue, we focused on the hippocampus, which might be a key region, and introduced some of these reports.

#### Interleukin-1 Receptor Antagonist

Prophylactic treatment with an IL-1 receptor antagonist significantly reduces plasma cytokines and hippocampal microgliosis and improves working memory impairment and cognitive dysfunction. There was no effect on high-mobility group box-1 levels (Terrando et al., 2010; Mina et al., 2014; Skelly et al., 2019).

#### Sulindac Sulfide

Administration of the anti-inflammatory agent, sulindac sulfide, at 3 weeks before infection suppressed LPS-induced amyloidogenesis, memory impairment, and neuronal cell death (Lee et al., 2008).

#### Nitric Oxide Synthase 2 Knockout Mice and Apocynin

Reactive free radicals NO and reactive oxygen species (ROS) are produced in sepsis. Previous reports have stated that nitric oxide synthase 2 (NOS2) is related to NO synthesis and that nicotinamide adenine dinucleotide phosphate oxidase 2 (Nox2) is involved in ROS synthesis. In a study using NOS2 knockout mice, LPS treatment resulted in sustained increased CD11b immunoreactivity in the hippocampus, frontal cortex, and cerebellum, with persistently increased glial activation in wild-type mice compared to that in NOS2 knockout mice. Expression analysis also showed that the brain mRNA levels of inflammatory factors were lower in LPS-treated NOS2 knockout mice than in wild-type mice and that no changes were observed in NOS2 knockout mice with respect to cognitive function (Weberpals et al., 2009).

In the cerebrum of cases with sepsis, microglia and astrocytes are activated and NO is produced; thus, resulting in apoptosis and necrosis in glial cells and neurons (Semmler et al., 2005).

NO induced by NOS2 may also cause neuroinflammatory responses and long-term changes in microglia and consequent cognitive dysfunction after LPS administration (Weberpals et al., 2009). Future studies are needed to clarify whether NO is detrimental to SAE and whether inhibition of NO may be neuroprotective (Semmler et al., 2005).

ROS produced by Nox2 cause oxidative damage to the hippocampus. This results in cognitive impairment after SAE and sepsis. Pharmacological inhibition of Nox2 (apocynin) or loss of Nox2 prevents glial cell activation, a central mechanism associated with SAE, suppressing oxidative stress in the hippocampus and preventing the development of long-term cognitive impairment (Hernandes et al., 2014).

#### Minocycline

Minocycline scavenges superoxide and peroxynitrite in non-neural *in vitro* assays (Whiteman and Halliwell, 1997; Zhang et al., 2003). Minocycline treatment reduces the cytokine levels and BBB decay in the hippocampus (Michels et al., 2015).

#### 7,8-Dihydroxyflavone

Brain-derived neurotrophic factor, a nerve growth factor, has a significant role in hippocampal LTP and experimental BDNF sequestration induces cognitive dysfunction (Figurov et al., 1996). Moreover, 7,8-dihydroxyflavone, a selective agonist of BDNF receptor tyrosine kinase B, administered to endotoxin-infected mice prevents memory loss and normalizes contextual fear conditioning by day 7 (Hippensteel et al., 2019).

#### Study Limitations

Based on these findings, we should further examine whether targeting other cytokine receptors for treatment can prevent or ameliorate cognitive decline in the future (Terrando et al., 2010). If these studies can be applied to clinical practice, it is expected that in the real world, the treatment of patients with sepsis will not only treat acute delirium but also prevent dementia in the chronic phase over the long term.

In this study, we primarily reviewed whether treatment intervention improves symptoms of SAE. It is also necessary to examine whether the intervention improves SAE and systemic inflammation. The focus of treatment of SAE remains proper management of systemic infections, sepsis, and systemic inflammatory response syndrome (Egi et al., 2021). Treatment of systemic infections could be considered to avoid substantial morbidity and mortality associated with SAE. It has been reported that LPS affected inflammation in the brain even in the absence of systemic IL-1β activity, although systemic IL-1β action was suppressed and cytokines in the blood were reduced when mice were treated with an IL-1 receptor antagonist in the LPS treatment model (Terrando et al., 2010; Skelly et al., 2019). Thus, although antagonists have some effect in suppressing systemic inflammation, they act only on part of the complex inflammatory cascade, and their effect may be limited. Further research is needed to expand the clinical applicability of these findings.

## Author Contributions

HI, SH, YK, HM, YI, and HO designed the study and wrote the manuscript. JO critically revised the manuscript for important intellectual content. All authors read and approved the final manuscript.

## Conflict of Interest

The authors declare that the research was conducted in the absence of any commercial or financial relationships that could be construed as a potential conflict of interest.

## Publisher’s Note

All claims expressed in this article are solely those of the authors and do not necessarily represent those of their affiliated organizations, or those of the publisher, the editors and the reviewers. Any product that may be evaluated in this article, or claim that may be made by its manufacturer, is not guaranteed or endorsed by the publisher.
